# Primary Hepatic Hemangiosarcoma With Hemoperitoneum in a Dog: Clinical Course and Therapeutic Outcome

**DOI:** 10.1155/crve/8709546

**Published:** 2026-01-28

**Authors:** Myeong-Yeon Lee, Jong-Hyun Moon, Jinsu Mok, Dowoo Lim, Ju-Yeong Kim, Seo-Yeong Jung, Eunbin Jeong, Hyomi Jang, Dong-In Jung

**Affiliations:** ^1^ Neuro Animal Medical Center, Busan, Republic of Korea; ^2^ Academic Committee, Korean Society of Feline Medicine (KSFM), Seoul, Republic of Korea; ^3^ College of Veterinary Medicine, Gyeongsang National University, Jinju, Republic of Korea, gnu.ac.kr

**Keywords:** chemotherapy, doxorubicin, receptor tyrosine kinase, toceranib, VAC protocol

## Abstract

A 14‐year‐old neutered male mixed‐breed dog presented with hemoperitoneum secondary to rupture of a primary hepatic mass. Following stabilization with whole blood transfusion, a hepatic lobectomy was performed. Histopathological examination and CD31 immunolabeling confirmed a diagnosis of hemangiosarcoma. Two weeks postoperatively, multiple intra‐abdominal metastases were identified. The multimodal therapeutic approach included one attempted VAC cycle (discontinued due to Grade 4 neutropenia), five cycles of single‐agent doxorubicin, and subsequent administration of toceranib with dose reduction for anemia. The dog survived 201 days after diagnosis. Postmortem extended immunohistochemistry demonstrated heterogeneous receptor tyrosine kinase (RTK) expression: EGFR and VEGFR positivity, strong membranous HER2 labeling in approximately 60% of tumor cells, low KIT expression, and negative PDGFR. This case underscores the aggressive metastatic nature of primary hepatic hemangiosarcoma following rupture and highlights practical considerations for systemic therapy selection and adverse event monitoring in clinical practice.

## 1. Introduction

Hemangiosarcoma (HSA), a mesenchymal neoplasm arising from vascular endothelial cells, accounts for approximately 2% of all canine tumors [1, 2]. Among these, almost half originate in the spleen, while other reported sites include the skin, heart, and lungs [1–4]. The liver is an uncommon primary site, comprising approximately 6% of all HSAs, although it is a frequent location for metastatic spread [1, 2]. In general, visceral HSAs, such as those from the spleen, heart, or liver, are considered more aggressive than cutaneous HSAs. This aggressiveness is attributed to early local invasion and a high propensity for early metastasis, primarily due to rupture of blood‐filled organs and subsequent hematogenous dissemination [1, 3, 5].

Owing to the fragile nature of the HSA tissue, rupture and hemorrhage are common; in addition, affected dogs often present with anemia, hypovolemia, weakness, or acute collapse [1, 4]. When the HSA occurs as a solitary lesion, surgical resection at the site of origin is considered the primary treatment option [1]. Computed tomography (CT) is recommended before surgery to assess tumor size, invasiveness, and the presence of metastasis [3]. However, considering the highly metastatic nature of HSA in dogs, chemotherapy is recommended in all cases, regardless of the metastatic status or surgical eligibility. Therefore, primary hepatic HSA should be managed with lobectomy following a CT scan, when feasible, followed by appropriate chemotherapy [1].

Among the chemotherapeutic options, doxorubicin, an anthracycline agent, has been reported to be the most effective, and most clinicians employ doxorubicin either as a single agent or in combination with other conventional drugs [1–4]. More recently, the expression of tyrosine kinase receptors such as platelet‐derived growth factor receptor (PDGFR), vascular endothelial growth factor receptor (VEGFR), and stem cell factor (SCF) receptor (KIT), has been identified in canine HSA tissues, leading to attempts at combining tyrosine kinase inhibitors (TKIs) such as toceranib with doxorubicin [1, 4, 6, 7]. In addition, the use of autologous cancer vaccines and peptide‐based anticancer vaccinations has been reported to prolong survival in dogs with HSA, although these remain in the clinical trial stage or are restricted in availability depending on the region [8–11].

Despite these therapeutic advances, HSA in dogs remains rare, and its prognosis is generally poor. A recent study reported a median survival time (MST) of 105 days in dogs with histopathologically confirmed HSA [12]. For splenic HSA, the reported MST after surgery ranges from 19 to 86 days, whereas surgery combined with doxorubicin‐based chemotherapy extends MST to approximately 5–7 months [1, 2, 4, 6, 13–16]. The HSAs arising in the heart, kidney, or retroperitoneal region have shown similarly poor outcomes [17, 18]. The MST of histopathologically confirmed primary hepatic HSA in dogs remains unclear. In a recent retrospective study that combined cases of primary and metastatic hepatic HSA, MST was reported to be approximately 79 days [12].

This case report describes a dog diagnosed with primary hepatic HSA that presented as an emergency due to a tumor rupture. The patient underwent a hepatic lobectomy and subsequent doxorubicin‐based chemotherapy and survived for 201 days. Although abdominal metastases involving the liver, mesentery, and spleen were detected within 2 weeks of rupture, thoracic metastases were not identified until death. In this case report, we aim to describe the treatment process, therapeutic outcomes, adverse effects, and prognosis of dogs with primary hepatic HSA.

## 2. Case Description

A 14‐year‐old neutered male mixed‐breed dog weighing 5.82 kg was referred to our hospital for an evaluation of lethargy, hypotension (systolic blood pressure, 70 mmHg), and anemia (hematocrit [HCT], 23%). The dog exhibited decreased activity and diarrhea for 2 weeks, followed by vomiting, anorexia, tachypnea, reduced water intake, and oliguria for the preceding 5 days. The patient had a 3‐year history of myxomatous mitral valve degeneration and was on cardiac medication. One month prior to presentation, analgesics were prescribed at a local veterinary clinic for lumbar pain.

On physical examination, the rectal temperature was 36.9°C, consistent with hypothermia. The capillary refill time was mildly prolonged at approximately 2 s, and the gingival mucous membranes appeared pale and dry. Systolic blood pressure was 120 mmHg at the time of examination; in addition, no abnormal heart or lung sounds were auscultated.

A complete blood count revealed anemia (HCT, 26.9%) and thrombocytopenia (platelets, 85 × 10^3^/*μ*L). Serum biochemical analysis showed hypoproteinemia (total protein, 5.0 g/dL) with reduced globulin concentration and mildly increased blood glucose and blood urea nitrogen levels. No significant abnormalities were observed (Table 1).

**Table 1:** Hematological parameters of the dog.

	**Initial visit**	**2 days postvincristine**	**37 days posttoceranib**	**Recurrent hemoperitoneum**	**Reference range**
Hematocrit (%)	26.9	48.6	27.8	19.6	37.3–61.7
Reticulocyte (k/*μ*L)	441.5	4.2	38.2	260.1	10–110
White blood cells (k/*μ*L)	14.56	1.08	6.54	22.34	5.05–16.76
Neutrophils (k/*μ*L)	11.42	0.39	4.91	17.54	2.95–11.64
Platelet (k/*μ*L)	85	102	127	39	148–484
Total protein (g/dL)	5.0	—	6.7	—	5.2–8.2
Albumin (g/dL)	3.5	—	3.2	—	2.2–4.4
Globulin (g/dL)	1.5	—	3.5	—	2.3–5.2
ALT (U/L)	32	—	38	—	10–140
ALP (U/L)	24	—	20	—	20–150
BUN (mg/dL)	40.7	—	46.6	—	7–27
Creatinine (mg/dL)	1.02	—	0.99	—	0.3–1.7

Abbreviations: ALP, alkaline phosphatase; ALT, alanine aminotransferase; BUN, blood urea nitrogen.

Thoracic radiography revealed no abnormalities. Abdominal radiography revealed decreased serosal detail, prompting abdominal ultrasonography. Ultrasonography revealed free abdominal fluid containing diffuse echogenic debris, heterogeneous parenchyma, and irregular right hepatic division margins (Figure 1). Approximately 160 mL of the ascitic fluid was collected for analysis. Grossly, the fluid appeared hemorrhagic, with a red blood cell count of 3.97 × 10^6^/*μ*L, total nucleated cell counts of 15.2/*μ*L, and total protein concentration of 4.8 g/dL, consistent with hemorrhagic effusion. The hemorrhage was suspected to have originated from the hepatic mass.

Figure 1Abdominal ultrasonography of the dog. (a) Irregular margins and heterogeneous parenchyma of the right hepatic division. (b) Free abdominal fluid containing diffuse echogenic debris.

**Figure:**
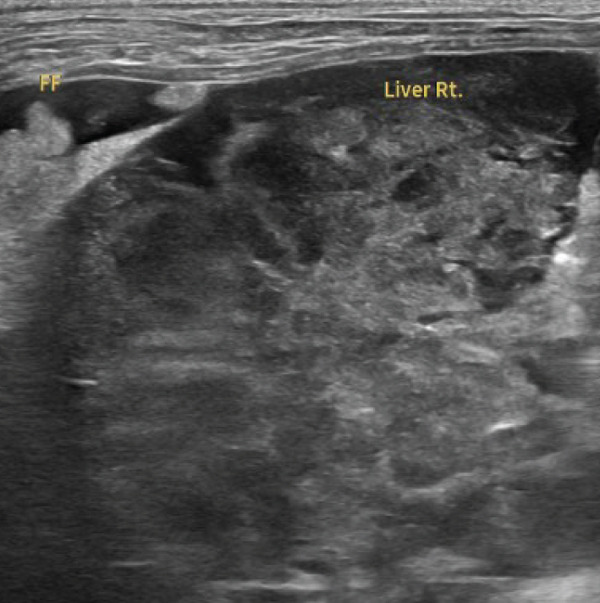
(a)

**Figure:**
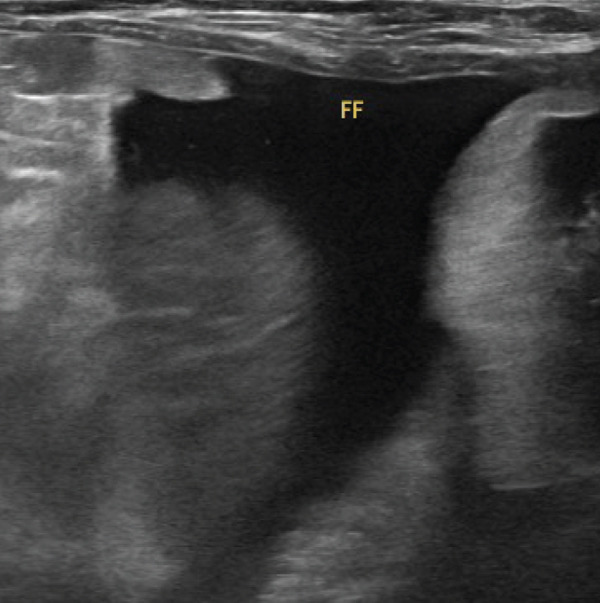
(b)

The patient was hospitalized and stabilized with a whole blood transfusion, intravenous fluids, and supportive medications including maropitant (1 mg/kg, IV, q24h), cefazolin (25 mg/kg, IV, q12h), and vitamin K1 (1 mg/kg, SC, q12h). Advanced diagnostic imaging and surgical resection of the hepatic mass were subsequently planned. Following transfusion, only minimal recurrence of hemorrhagic effusion was observed, and the patient′s condition stabilized with an improvement in HCT level (32.4%) and normalization of body temperature.

Echocardiography was performed to evaluate anesthetic risk, revealing mild thickening and prolapse of the mitral valves. The left atrial‐to‐aortic root ratio was 1.52, the left ventricular internal diameter in diastole was 1.82 cm, and the mitral regurgitant jet velocity was 5.69 m/s. These findings were consistent with ACVIM Stage B2 myxomatous mitral valve disease, for which oral pimobendan (0.25 mg/kg, PO, q12h) was prescribed.

Subsequently, thoracic and abdominal CT were performed under general anesthesia (December 28). No abnormalities were identified on thoracic CT, while the abdominal CT demonstrated a large, irregularly marginated mass with a “rumpy‐bumpy” contour, arising from the right lateral hepatic lobe measuring 94 × 63 × 54 mm. The mass exhibited heterogeneous contrast enhancement with central hypodensity and multiple vascularized areas. The lesion extended caudally, abutting the right kidney, compressing the caudal vena cava, and impinging ventrally into the right pancreatic lobe. Dorsally, indistinct margins with evidence of capsular disruption suggested a rupture (Figure 2).

Figure 2(a) Transverse and (b) dorsal postcontrast CT images of the dog. A large amorphous mass arising from the right lateral hepatic lobe shows central hypodense areas and multiple regions of vascularization.

**Figure:**
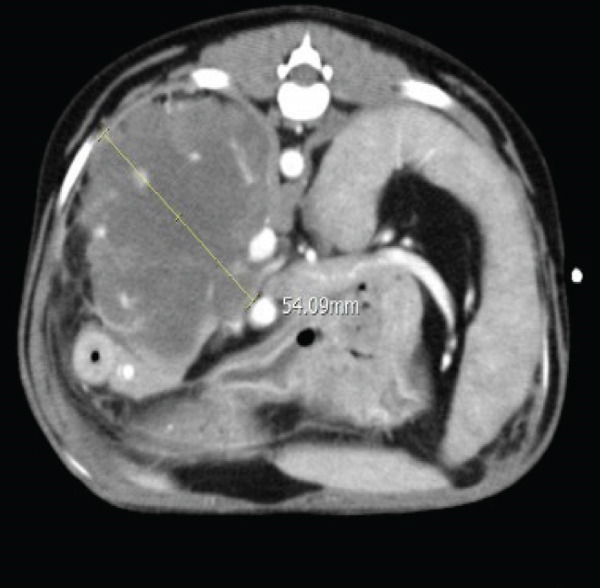
(a)

**Figure:**
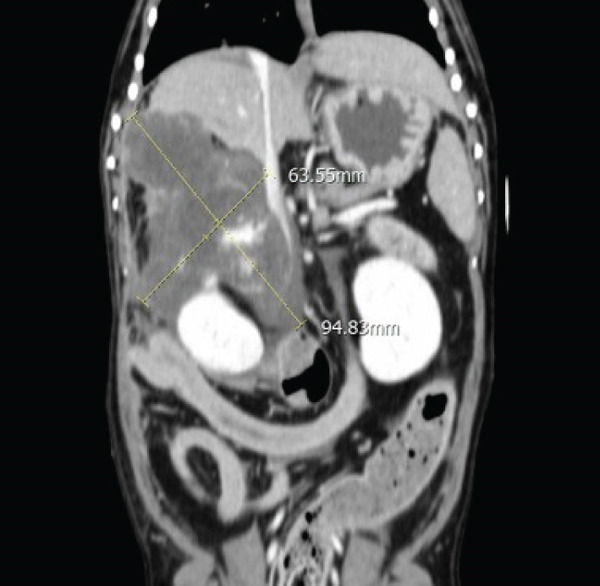
(b)

Based on the CT findings, the abdominal hemorrhage was attributed to the rupture of a primary hepatic tumor, and hepatic lobectomy was performed. A ventral midline laparotomy was performed, and exploratory examination revealed a dark red, irregularly surfaced mass originating from the right lateral hepatic lobe. No additional nodules or masses suggestive of neoplasia were identified in the liver or abdominal cavity. Numerous blood clots were observed within the mesentery and peritoneal cavity, which were consistent with a hemorrhage from the hepatic mass.

The central hepatic division and the duodenum were retracted to expose the right lateral lobe. The adjacent tissues and right triangular ligament were dissected using a vessel‐sealing device (LigaSure). During this procedure, an additional hemorrhage occurred, leading to systemic hypotension, which necessitated concurrent whole blood transfusion. The suspected local invasiveness of the mass suspected on CT was not confirmed intraoperatively. Instead, the tumor was compressive, with only mild adhesions to the surrounding tissues, allowing for a relatively straightforward separation from the adjacent organs.

The hepatic vein, artery, and the bile duct supplying the affected lobe were ligated using polymer hemoclips (DMD Poly‐Lok; MedNet GmbH, Germany), and the entire right lateral lobe was excised. A minor hemorrhage from the transection margin was controlled using a topical hemostatic agent. After evacuation of the intra‐abdominal blood clots, the abdominal cavity was thoroughly lavaged, and routine abdominal wall closure was performed (Figure 3).

Figure 3(a) Intraoperative view of the hepatic lobectomy and (b) the resected right lateral hepatic mass in the dog.

**Figure:**
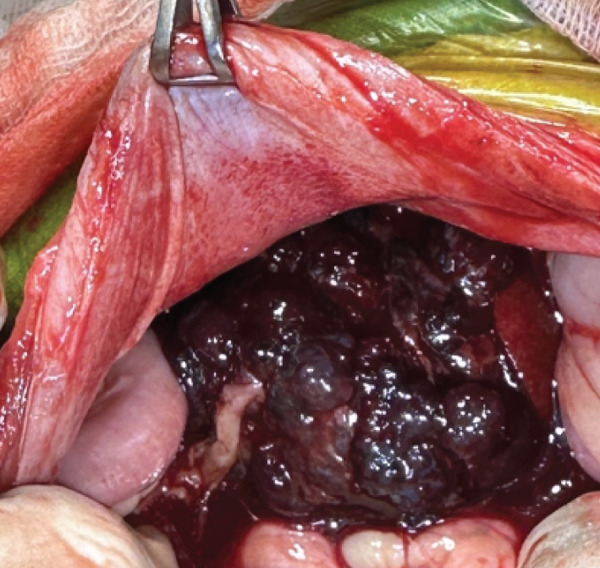
(a)

**Figure:**
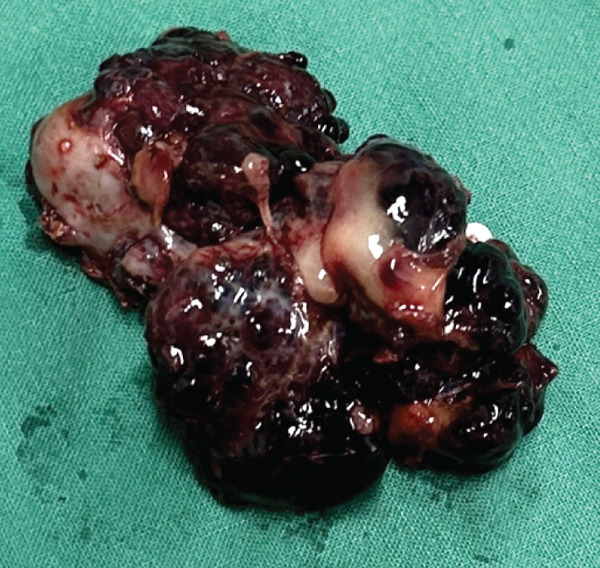
(b)

Histopathological examination revealed pleomorphic round‐to‐polygonal neoplastic cells with pale eosinophilic cytoplasm and variably distinct borders forming irregular vascular channels supported by delicate collagenous trabeculae (IDEXX Laboratories, Westbrook, Maine). Nuclei were round to ovoid or irregular in shape, with finely stippled to marginal chromatin and one or more prominent nucleoli. Marked anisokaryosis and anisocytosis were evident, and 14 mitotic figures were observed within 10 consecutive high‐power fields (Figure 4a,b).

Figure 4Histopathological and immunohistochemical findings of the tumor. (a, b) Irregular vascular channels lined by pleomorphic neoplastic cells with marked anisocytosis and anisokaryosis, and streams with collagenous stroma are observed (H&E stain): (a) 10× magnification and (b) 40× magnification. (c, d) Neoplastic cells show positive immunoreactivity for CD31: (c) 10× magnification and (d) 40× magnification. Based on these features, the tumor was diagnosed as hemangiosarcoma.

**Figure:**
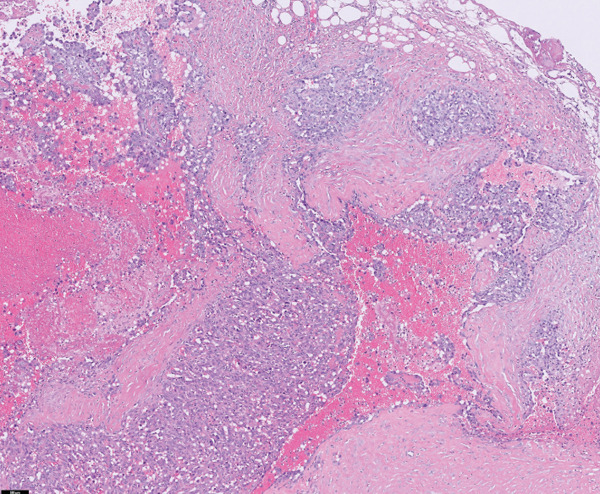
(a)

**Figure:**
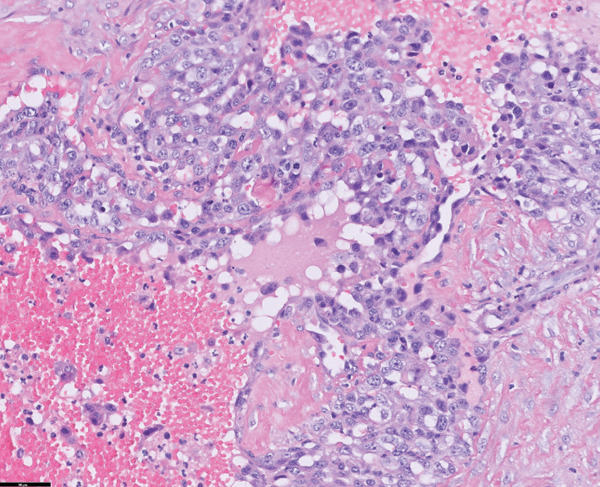
(b)

**Figure:**
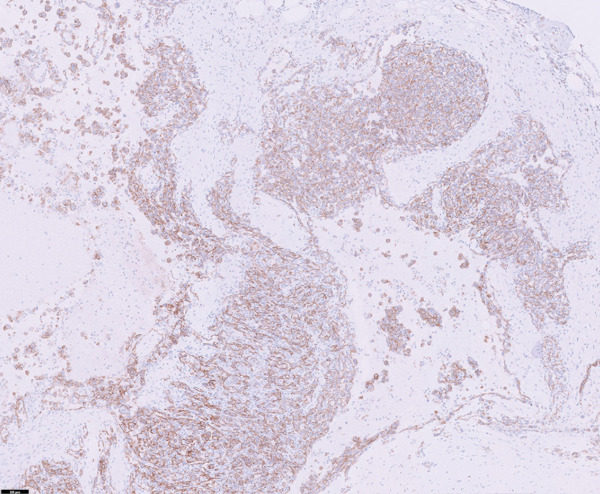
(c)

**Figure:**
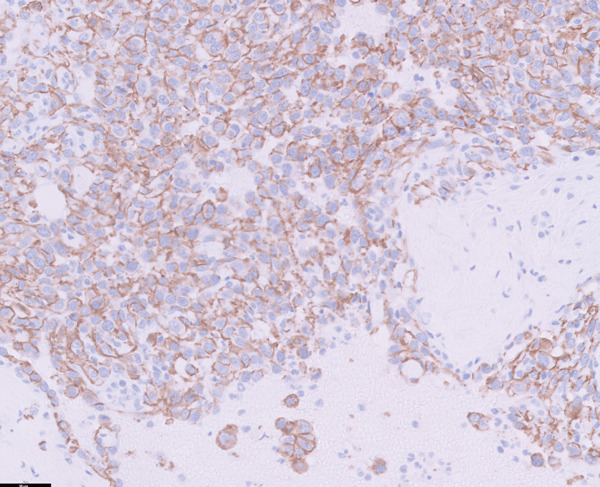
(d)

Immunohistochemical staining demonstrated positive immunoreactivity for endothelial cell–specific markers, CD31, and together with the histopathological features, these findings confirmed a diagnosis of HSA.

Based on the imaging findings, gross surgical observations, and histopathology, the patient was diagnosed with Stage II primary hepatic HSA. Abdominal ultrasonography performed 2 weeks postoperatively revealed multiple mesenteric nodules. A week later, a heterogeneous parenchymal mass was detected at the mesentery, hepatic lobectomy site, and subsequent evaluation identified nodules in the spleen, raising the suspicion of metastatic disease (Figures 5a, 5b, and 5c).

Figure 5Abdominal ultrasonography demonstrating multiple metastatic lesions: (a) mesenteric nodules, (b) a heterogeneous parenchymal mass at the hepatic lobectomy site (white arrow), (c) splenic nodules (yellow arrow), and (d) a lesion caudal to the urinary bladder (large‐headed yellow arrow).

**Figure:**
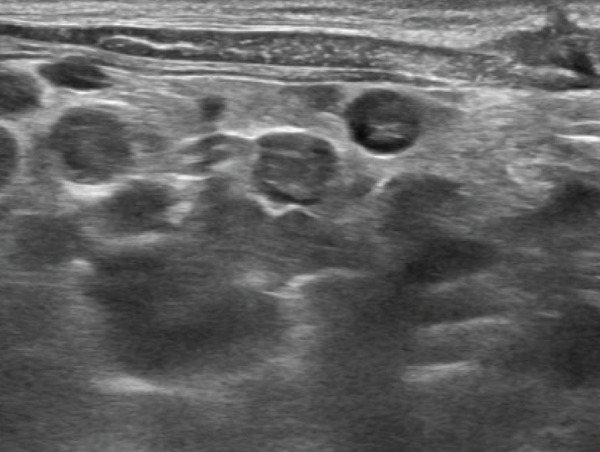
(a)

**Figure:**
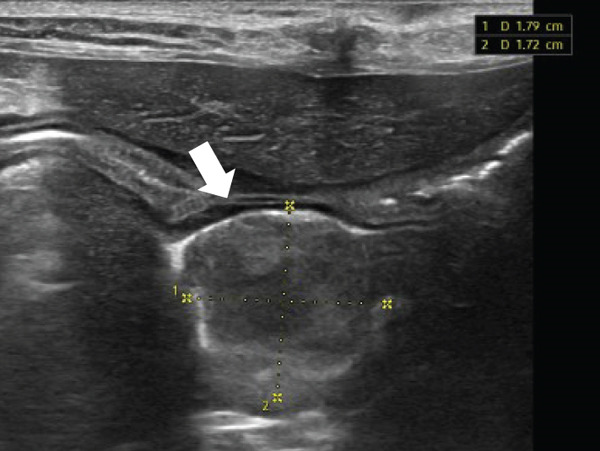
(b)

**Figure:**
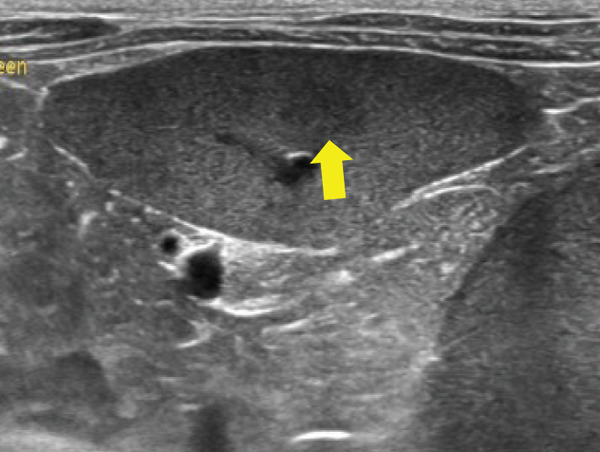
(c)

**Figure:**
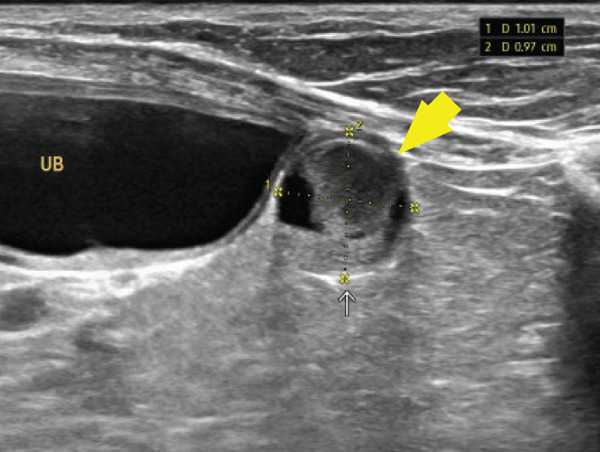
(d)

Given these findings, an aggressive chemotherapy regimen was initiated using the vincristine, doxorubicin, and cyclophosphamide (VAC) protocol (Table 2). Following a previously reported VAC protocol [19], the treatment plan consisted of doxorubicin (1 mg/kg, IV) on Day 1, vincristine (0.7 mg/m^2^, IV) on Day 8, and cyclophosphamide (250 mg/m^2^, PO) on Day 10. However, the day after vincristine administration, the patient developed severe vomiting, anorexia, and bone marrow suppression. Abdominal ultrasonography revealed a lesion caudal to the urinary bladder, raising the suspicion of further metastasis (Figure 5d). Consequently, propranolol (0.3 mg/kg, PO, q12h) was added to the protocol, and cyclophosphamide (250 mg/m^2^, IV) was administered 10 days after the second chemotherapy cycle (Table 1).

**Table 2:** Chemotherapy treatment schedule for dogs.

	**Day 1**	**Day 8**	**Day 15**	**Day 18**	**Day 25**	**Day 46**	**Day 67**	**Day 88**	**Day 109**	**Day 130**	**Day 167**
Doxorubicin (1 mg/kg, IV)	•				•	•	•	•	•		
Vincristine (0.7 mg/m2, IV)		•									
Cyclophosphamide (250 mg/m2, IV)				•							
Propranolol (0.3‐0.75 mg/kg, PO, q 12 h)			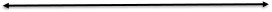								
Toceranib (2.5‐2.6 mg/kg, PO, MWF)										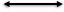	

MWF = Monday–Wednesday–Friday

However, owing to the owner′s refusal to continue vincristine administration after the patient experienced severe adverse effects, the protocol was modified to a single‐agent doxorubicin regimen (1 mg/kg, IV, q3 weeks) with propranolol increased to 0.5 mg/kg (PO, q12h) (Table 2). During four cycles of single‐agent doxorubicin, intermittent vomiting and diarrhea were observed, but the patient′s overall condition remained stable. Abdominal ultrasonography revealed a reduction in the size of some metastatic lesions, although the number of detectable metastatic foci gradually increased. Five doxorubicin cycles were administered. Throughout the treatment, the patient maintained a good general condition, and only mild leukopenia was observed without other clinically significant bone marrow suppression.

At the owner′s request, chemotherapy was subsequently switched from cytotoxic agents to targeted therapy with toceranib, administered at 15 mg/dog (2.6 mg/kg, PO, Monday–Wednesday–Friday schedule), while propranolol therapy was continued as described (Table 2). Thirty‐six days after initiating toceranib, the patient developed mild anemia (HCT, 35.1%), thrombocytopenia (platelets, 122 × 10^3^/*μ*L), and a small volume of hemorrhagic ascites. Toceranib was therefore dose‐reduced to 2.5 mg/kg (PO, MWF), and propranolol was increased to 0.75 mg/kg (PO, q12h). Despite these adjustments, nonregenerative anemia persisted, and toceranib was discontinued on Day 37 (Table 1).

Over the following 2 weeks, the patient required two whole blood transfusions; however, clinical recovery was not achieved. On Day 179 of chemotherapy (Day 201 after the initial presentation), progressive hemoperitoneum was documented (Table 1). Two days later, euthanasia was performed at the owner′s request.

Following the patient′s death, archived tumor tissue samples retained in the pathology laboratory (IDEXX Laboratories, Westbrook, Maine) were subjected to additional immunohistochemical evaluation of receptor tyrosine kinase (RTK) expression to investigate the basis for the poor response to toceranib (Table 3). The analysis revealed that neoplastic cells did not label for vascular endothelial growth factor (VEGF) or PDGFR. Approximately 10% of neoplastic cells were positive for KIT, and 50% demonstrated positivity for SCF. Strong membranous labeling for human epidermal growth factor Receptor 2 (HER2) was observed in approximately 60% of neoplastic cells, corresponding to a 3 score of stain intensity, which is interpreted as positive. Moreover, 100% of the neoplastic cells were positive for both epidermal growth factor receptor (EGFR) and VEGFR.

**Table 3:** Tyrosine kinase receptor antibodies for immunohistochemistry of the hepatic tumor sample from this dog.

**Target**	**Clonality**	**Dilution**	**Vendor**	**% staining of cells**
EGFR	Rabbit polyclonal	1:100	Biorbyt	100
HER2	Rabbit monoclonal	—	Ventana	60
KIT	Rabbit polyclonal	1:400	Dako	10
PDGFR	Rabbit polyclonal	1:100	Santa Cruz	0
SCF	Mouse monoclonal	1:100	Santa Cruz	50
VEGF	Mouse monoclonal	1:100	Abcam	0
VEGFR	Mouse monoclonal	1:300	Santa Cruz	100

Abbreviations: EGFR, epidermal growth factor receptor; HER2, human epidermal growth factor Receptor 2; PDGFR, platelet‐derived growth factor receptor; SCF, stem cell factor; VEGF, vascular endothelial growth factor; VEGFR, vascular endothelial growth factor receptor.

## 3. Discussion

This case report presents a dog with primary hepatic HSA with rupture, diagnosed by diagnostic imaging, histopathology, and immunohistochemistry (IHC), and managed through surgical and medical approaches. Primary hepatic HSA in dogs, which accounts for approximately 6% of canine HSAs, is typically managed through surgical and medical approaches, yet detailed reports describing its diagnostic process and treatment course remain limited in the veterinary literature [1, 2].

Most reports of canine hepatic HSA are included within case series of HSAs from other organs or do not distinguish between primary and metastatic hepatic masses, with treatment methods, clinical courses, and prognoses described predominantly for splenic or cardiac HSAs [1–4, 12, 16–18, 20–22]. To date, only a few reports have specifically documented cases in which HSA is confined to the liver at the time of diagnosis [23, 24]. In one such report, the patient died 7 days after surgical excision, as treatment was discontinued at the owner′s request, in contrast to the present case, in which the dog survived for 201 days with aggressive chemotherapy [23]. Another report briefly mentioned the treatment process of primary hepatic HSA, but only in the context of a larger case series with limited details [24].

In humans, primary hepatic HSA progresses rapidly and is rarely amenable to surgical resection at the time of diagnosis, with one study of 11,939 cases reporting that all patients already had multiple systemic metastases precluding surgery, and another indicating that fewer than 20% of patients were able to undergo surgical treatment [25–27]. A clinical course of a ruptured primary hepatic HSA has been reported despite an attempt at surgical excision of the tumor, in which a new metastatic liver mass was detected 21 days after surgical resection. The lesion enlarged within 6 days, and the patient died 36 days after surgery [28]. The prognosis of primary hepatic HSA remains dismal, with most patients dying within 1 year in human medicine [27].

Similarly, in a canine case report, metastases to the diaphragm and mesentery were identified at necropsy 7 days after surgery for primary hepatic HSA, although it was not detected at that time of diagnosis [23]. This report suggested that metastasis was likely already established at the time of surgical resection, because the diagnosis was made using radiography and abdominal ultrasonography without performing a CT scan [23]. In the present case, diagnostic imaging (radiography, abdominal ultrasonography, and CT) and gross surgical inspection revealed no evidence of metastasis at the time of diagnosis. However, 2 weeks later, multiple suspected metastatic nodules were detected in the mesentery, liver, and spleen. A full postmortem necropsy was recommended for accurate systemic assessment of metastatic progression and outcome; however, it was not performed due to the client′s refusal. This limitation has been acknowledged in this case.

A previous study reported that in dogs with splenic HSA ruptures managed with surgical resection followed by doxorubicin‐based and metronomic chemotherapy, 3 out of 10 dogs developed metastatic lesions, with the time of detection at 119, 151, and 460 days after treatment initiation [29]. To date, no published study has specifically addressed the timing of metastasis following the rupture of primary hepatic HSA in dogs. Therefore, additional case reports and larger studies focusing exclusively on primary hepatic HSA are needed to clarify its clinical behavior, prognosis, and the timing of metastasis.

In the present case, as multiple metastatic lesions were detected in the abdominal cavity shortly after the lobectomy, an advanced‐stage HSA protocol was selected using a doxorubicin‐based VAC regimen [16, 19]. However, due to the severe adverse effects of vincristine (VCOG‐CTCAE V2 Grade 4 neutropenia and Grade 2 vomiting and anorexia), only one cycle could be administered, preventing a meaningful assessment of its antitumor efficacy [30]. Subsequently, five cycles of single‐agent doxorubicin administered every 3 weeks resulted in only mild adverse effects, including Grade 2 diarrhea, Grade 1 vomiting, and mild leukopenia.

These outcomes are consistent with previous reports on canine HSA, in which doxorubicin, either alone or in combination with other drugs, yielded limited survival benefits and nearly all patients ultimately succumbed to the disease [6, 15, 16, 31, 32]. In splenic HSA, the MST of dogs treated with surgery alone ranged from 19 to 86 days, whereas surgery combined with doxorubicin‐based chemotherapy extended the MST to only 5–7 months [1, 2, 13, 14]. In dogs with presumptive cardiac HSA, the biological response rate to doxorubicin was reported to be 68%; however, the MST remained poor, with untreated dogs surviving a median of 66 days compared to 116 days in treated dogs [33]. Similarly, in the present case, the administration of doxorubicin and other chemotherapeutic agents resulted in only temporary slowing of disease progression, without durable control. However, considering the rapid progression of metastasis after surgery, the 201‐day survival suggests that multimodal therapy may provide a clinically meaningful extension of survival for patients with primary hepatic HSA. Further studies are needed to establish evidence‐based treatment strategies for this rare neoplasm.

Toceranib, a TKI, was subsequently administered; however, it also failed to halt disease progression in this case. This outcome is consistent with a previous study in which toceranib was used as maintenance therapy following doxorubicin in 31 dogs with splenic HSA, where it did not confer any improvement in either disease‐free interval or overall survival [6].

Several studies have reported the expression of RTKs in canine HSA tissues [1, 4, 6, 7, 34–37]. One study demonstrated variable RTK expression in canine hemangiomas (HAs) and HSAs, including 29 visceral HSAs and one case of primary hepatic HSA, although differences in expression according to the anatomical location of the tumors were not reported [36]. In that study, the SCF receptor KIT (CD117) was negative in benign HAs, whereas in HSAs, KIT expression was variably positive, ranging from low to high levels [36]. Another study also found that KIT expression in canine splenic and metastatic hepatic HSA tissues was slightly higher than in normal splenic tissue, although only at a minimal level [35]. In the tumor tissue of the present case, KIT showed low‐level positivity of approximately 10%, whereas its ligand, SCF, showed about 50% positivity. These findings suggest that TKIs primarily targeting KIT may have inconsistent efficacy in canine HSA.

In the study of 29 visceral HSAs, VEGFR, which is upregulated in endothelial cells of tumor neovasculature, was expressed in most HSAs and at higher levels than in HAs, whereas its ligand VEGF was not overexpressed [36]. Similarly, in the present case, VEGFR was 100% positive, while VEGF was negative, consistent with previous studies. In addition, PDGFR was negative, whereas EGFR was 100% positive.

RTKs such as KIT, VEGFR, PDGFR, and EGFR have been investigated as potential therapeutic targets for the application of various TKIs in canine HSA; however, their expression levels have been inconsistent, and no clear association with clinical outcomes has been established [6, 34, 36, 37]. Therefore, despite the expression of various RTKs, no TKI has yet been proposed as a critical treatment option for canine HSA, and further studies are warranted to elucidate the scientific basis for the lack of significant antitumor effects of toceranib observed in previous reports and in the present case.

In the present case, HER2, another RTK, showed approximately 60% positivity with strong labeling on the tumor cell membranes. The expression of HER2 has been investigated in epithelial‐origin tumors such as canine mammary gland tumors and is known to be associated with factors such as tumor grade and stage facilitating cell proliferation [38–42]. To date, no studies have investigated HER2 expression in canine sarcoma tissues. In one study that reported the expression levels of HER2 in normal hepatoid tissues, hepatoid gland adenomas, hepatoid gland epitheliomas, and hepatoid gland carcinomas, HER2 was found to be negative in normal hepatoid tissues and hepatoid gland adenomas and exclusively positive in hepatoid gland epitheliomas and hepatoid gland carcinomas [43]. Based on this, it is considered that some cells of the primary hepatic HSA in the present case may have exhibited carcinomatous or epithelioma‐like features. On the other hand, as this receptor is known to be expressed in some normal human epithelial cells, albeit at lower levels than in tumor tissues [44], further studies are needed to investigate its expression within the panel of RTK IHC in canine liver tissue or HSA.

Propranolol, a nonselective *β*‐adrenergic receptor antagonist, has been reported to exert antiproliferative and cytotoxic effects against tumors of vascular endothelial origin [45–50]. In veterinary oncology, propranolol has been used in dogs with HSA, although the results have been variable without dramatic antitumor effects [24, 51]. However, one study reported that dogs younger than 6 years with splenic HSA achieved longer survival when propranolol was combined with doxorubicin compared with doxorubicin alone, suggesting that this effect may be related to age‐dependent drug metabolism [51]. In the present case, propranolol could not be applied as an adjunctive chemotherapeutic agent from the outset, and the absence of a control group precluded confirmation of any overall survival benefit. Nevertheless, propranolol was well tolerated and associated with minimal adverse effects in this patient, indicating its potential role as an adjunctive agent in the management of canine HSA.

This case report highlights the diagnostic approach, surgical resection, and chemotherapy used in a dog with primary hepatic HSA, providing meaningful insights into the biological and clinical behavior of this rare tumor. Primary hepatic HSA is an uncommon neoplasm in both veterinary and human medicine; however, veterinary reports focusing specifically on canine primary hepatic HSA remain scarce. Further studies are warranted to better characterize clinical features, therapeutic strategies, treatment responses, adverse effects, and the overall prognosis.

## Ethics Statement

This case report was exempt from ethics approval, as it involved the retrospective analysis of a single patient′s clinical data and did not involve any experimental procedures. Informed consent for the use of the dog′s medical information was obtained from the owner.

## Disclosure

All authors reviewed and approved the submitted manuscript.

## Conflicts of Interest

The authors declare no conflicts of interest.

## Author Contributions

All authors contributed to the study, including planning, case accrual, algorithm development, and writing of the manuscript.

## Funding

No funding was received for this manuscript.

## Data Availability

The data that support the findings of this study are available on request from the corresponding authors. The data are not publicly available due to privacy or ethical restrictions.
